# Success Rates and Adverse Events of Advanced Airway Management Techniques in the Emergency Department: A Prospective Observational Study

**DOI:** 10.7759/cureus.117660

**Published:** 2026-10-08

**Authors:** Jithin Suresh, Ramu R J, Anand K V, Jithin Varghese, Nikhil Paul

**Affiliations:** 1 Emergency Medicine, Government Medical College Kannur, Pariyaram, IND; 2 Emergency Medicine, Sree Gokulam Medical College and Research Foundation, Thiruvananthapuram, IND; 3 Emergency Medicine, Muslim Educational Society (MES) Medical College and Hospital, Malappuram, IND; 4 Emergency Medicine, Kasturba Medical College, Mangalore, IND

**Keywords:** advanced airway management, adverse events, awake intubation, crash intubation, delayed sequence intubation, emergency department, first-pass success, rapid sequence intubation

## Abstract

Background: Advanced airway management is a core component of the emergency response. Different intubation techniques may differ in their likelihood of success on the first attempt and in the incidence of associated adverse events. Analyzing routine emergency practice can identify opportunities to improve airway safety and procedural performance.

Objective: This study aimed to determine the first-pass success and complications of different advanced airway-management methods and to describe the indications for, and medications used in, emergency airway management.

Methods: This prospective observational study enrolled adult patients requiring advanced airway management during an emergency department (ED) visit over 12 months (December 2019 to November 2020) at a tertiary care teaching hospital in Kannur, Kerala, India. Of 247 patients assessed, three were excluded because consent could not be obtained, leaving 244 patients. Airway techniques were classified as rapid sequence intubation (RSI), delayed sequence intubation (DSI), crash intubation, and awake intubation. All intubations were performed by emergency physicians with prior experience of more than 100 endotracheal intubations. The primary outcomes were first-pass success and adverse events occurring within 15 minutes of airway management. Proportions are reported with 95% confidence intervals (CIs) calculated by the Wilson score method, and associations were assessed using the Fisher-Freeman-Halton exact test.

Results: The mean age of participants was 63.1 ± 9.7 years, and 136 (55.7%) were male. RSI was the most frequently used technique (179/244, 73.4%), followed by crash intubation (38/244, 15.6%), DSI (25/244, 10.2%), and awake intubation (2/244, 0.8%). Overall first-pass success was 91.4% (223/244; 95% CI: 87.2-94.3), and all 244 patients were ultimately intubated within two attempts, with no surgical airway required. First-pass success was high across the cohort, although comparisons between techniques were inconclusive because of markedly unequal subgroup sizes, particularly the very small awake-intubation group. Adverse events occurred in 30 patients (12.3%; 95% CI: 8.7-17.0), comprising 8.9% of RSI, 28.0% of DSI, and 18.4% of crash intubation cases, with none among the two awake-intubation cases (p = 0.031). The most common adverse event was dental trauma. Non-traumatic indications predominated (75.0%), the leading indication being respiratory failure, whereas head injury was the most common traumatic indication. Etomidate was the most frequently used induction agent, and rocuronium was the only neuromuscular blocking agent recorded.

Conclusions: Advanced airway management in this ED cohort was associated with a high first-pass success rate and a relatively low rate of adverse events recorded within the 15-minute observation window, with RSI the predominant technique and all intubations performed by experienced operators. The study was designed to estimate the overall first-pass success rate, not to compare techniques: subgroups were markedly unequal, technique was selected on clinical grounds rather than randomly allocated, and pre-intubation physiological severity was not recorded. The apparent differences in first-pass success and adverse events among techniques are therefore inconclusive and are likely to reflect confounding by indication; no inference about the relative effectiveness or safety of individual techniques should be drawn from these data.

## Introduction

Emergency airway management is one of the most essential and frequently performed procedures in emergency medicine, and it remains a complex, high-stakes task in critically ill patients [1]. If a patent airway is not established and maintained, inadequate gas exchange can progress to ventilatory failure, hypoxic injury, cardiovascular collapse, and death. Patients requiring emergency intubation frequently have markedly deranged physiology-respiratory failure, depressed consciousness, trauma, shock, or cardiac arrest that makes airway management considerably more difficult than elective, controlled intubation [1]. Large multicenter airway registries show that most emergency department (ED) intubations are performed by emergency physicians, with rapid sequence intubation (RSI) among the most frequently used methods for definitive airway control [1,2].

The principal goal of emergency tracheal intubation is to secure the airway while minimizing adverse peri-intubation physiological effects. In patients with limited physiological reserve, airway manipulation, sedatives, neuromuscular blockade, apnea, and positive-pressure ventilation may precipitate hypoxemia, hypotension, arrhythmia, or cardiac arrest; the international INTUBE study, which enrolled 2,964 critically ill patients across 197 sites in 29 countries, found that at least one major adverse event occurred around the time of intubation in 45.2% of patients [3].

First-pass success is a key quality measure in airway management. Its clinical relevance lies in the fact that additional laryngoscopy attempts carry a higher risk of complications: in the INTUBE cohort, major peri-intubation adverse events were less frequent with first-pass success than when two attempts (43.2% vs 51.5%) or three or more attempts (43.2% vs 58.0%) were required [3]. Optimizing the first attempt has therefore become central to contemporary emergency airway care.

The choice of technique depends on the patient's physiology, airway anatomy, and level of consciousness and the urgency of intervention. RSI involves the administration of an induction agent immediately followed by a neuromuscular blocking agent to achieve rapid tracheal intubation and is commonly used when a definitive airway is required urgently and there is no significant contraindication to paralysis. Current clinical practice guidelines continue to address optimization of oxygenation, medication selection, neuromuscular blockade, and peri-intubation physiology during RSI of the critically ill adult [4].

For agitated or uncooperative patients who cannot tolerate adequate preoxygenation, delayed sequence intubation (DSI) is an alternative. DSI uses a dissociative dose of sedation (typically ketamine) to permit adequate preoxygenation before paralysis and intubation. In a randomized controlled trial of critically injured patients who could not be adequately preoxygenated because of agitation, DSI significantly reduced peri-intubation hypoxia (8.0% vs 35.0%) and improved first-attempt success (83.0% vs 69.0%) compared with RSI [5].

Other scenarios call for different approaches. Crash intubation is used in the profoundly unresponsive patient, for example, in cardiac arrest or another immediately life-threatening situation, where airway control cannot be delayed. Awake intubation, by contrast, maintains spontaneous ventilation and may be useful when a difficult airway is anticipated; its essential principles include appropriate patient selection, adequate topical anesthesia, cautious sedation, confirmation of tube placement, and avoidance of repeated attempts [6]. Awake intubation is, however, rarely performed in the ED: in an analysis of the National Emergency Airway Registry, it was used in only 0.4% of more than 19,000 ED intubation encounters [7].

Although each technique is well described individually, practice varies with patient factors, indication for airway intervention, operator experience, medication availability, and local protocols [1]. Assessing the real-world outcomes and complications of the different approaches is therefore valuable for identifying opportunities to improve emergency airway care, particularly in settings where prospective comparative data are limited.

Aim and objectives

The main objective of this study was to assess the success rates and adverse events of advanced airway-management techniques in adult patients requiring emergency airway management. The specific objectives were to (1) determine the first-pass success rates of RSI, DSI, crash intubation, and awake intubation; (2) evaluate the frequency and pattern of adverse events associated with these techniques; (3) describe the traumatic and non-traumatic indications for emergency airway management; and (4) characterize the induction and neuromuscular blocking agents used.

## Materials and methods

Study design and duration

This was a prospective observational study of adult patients who underwent advanced airway management in a tertiary care ED. The study was conducted at the Academy of Medical Sciences (ACME), Pariyaram, Kannur, Kerala, India, with patients recruited prospectively over one year (December 2019 to November 2020). The average annual ED attendance during the study period was approximately 45,000. The study is reported in accordance with the Strengthening the Reporting of Observational Studies in Epidemiology (STROBE) guidelines, and the flow of participants through the study is shown in Figure 1.

**Figure 1:**
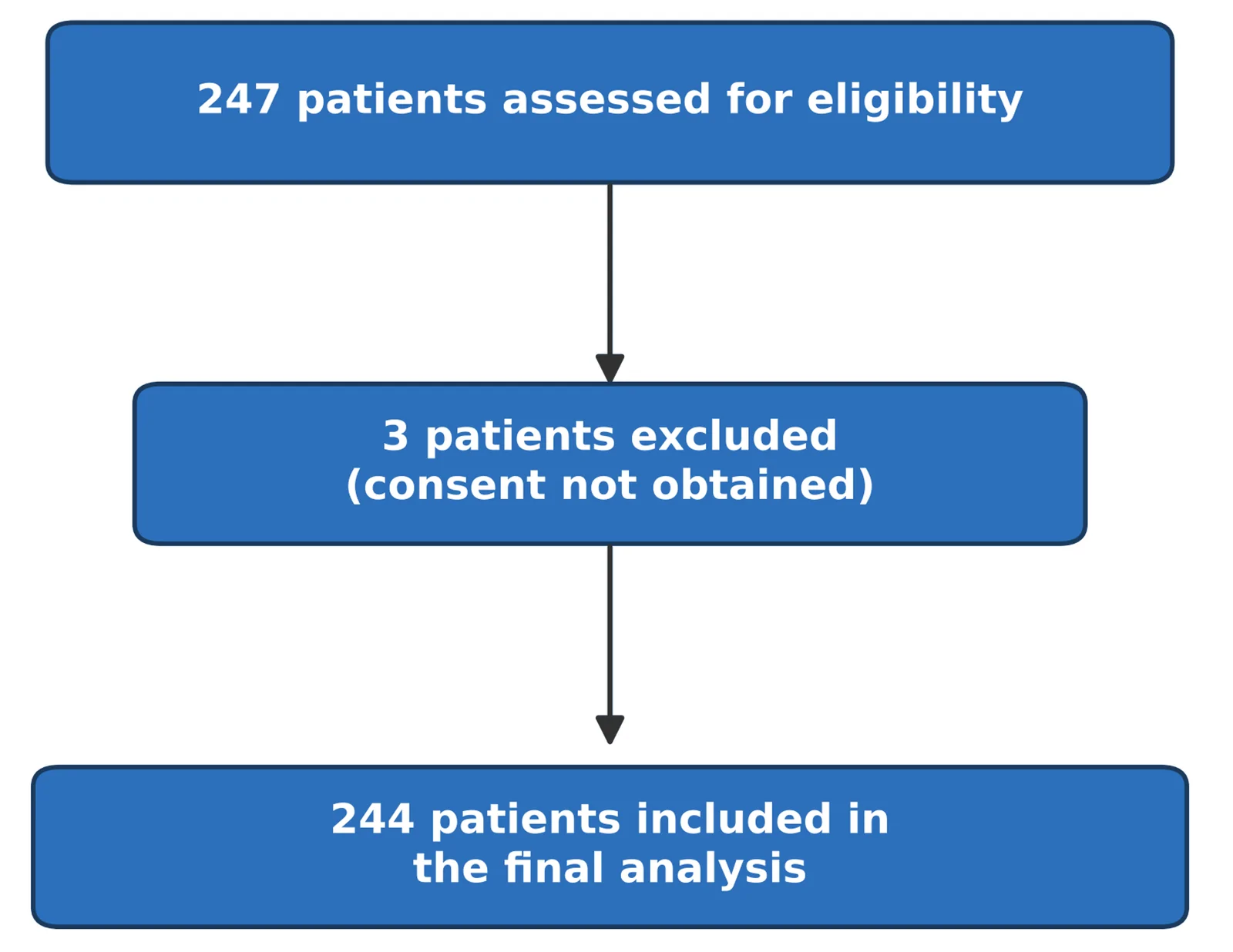
Flow of patients through the study (STROBE flow diagram) Two hundred and forty-seven patients were assessed for eligibility, three excluded because consent could not be obtained, and 244 included in the final analysis. STROBE: Strengthening the Reporting of Observational Studies in Epidemiology

The study period overlapped the first year of the COVID-19 pandemic in India, which may have influenced ED attendance, the case mix of intubation indications, the use of personal protective equipment, preoxygenation and airway practice, and operator availability. Emergency airway management continued to follow standard institutional principles throughout. Pandemic-related protocol deviations such as designated senior airway operators, modified preoxygenation practices, and aerosol-containment measures were not uniformly tracked in the study dataset, and their influence on device selection and procedural timing could therefore not be quantified.

Study Population and Sample Size

The study population comprised adult patients presenting to the ED who required emergency advanced airway intervention. The minimum sample size was calculated using the formula $\mathrm{N}=4\mathrm{pq}/\mathrm{d}^{&sup2;}$, where p was the expected success rate (83.0%) based on previously published data [8], q the failure rate (17.0%), and d the precision (5.0%). This yielded a minimum required sample of 226 patients. Of 247 patients initially considered, three were excluded because consent could not be obtained, resulting in a final study population of 244 patients.

Eligibility Criteria

Patients aged 18 years or older who presented to the ED and required emergency advanced airway management were eligible for inclusion. Patients were excluded if they were younger than 18 years and already had an advanced airway in place at presentation or if consent for participation could not be obtained.

Emergency Airway Assessment and Management

During the primary assessment in the ED, patients who required airway intervention were identified. The decision for definitive airway management was based on the clinical situation, including inadequate airway protection, inadequate oxygenation and/or ventilation, or anticipated clinical deterioration. The appropriate airway-management technique was selected according to the clinical condition and the airway-management needs of the individual patient. Airway interventions were performed by emergency physicians with substantial airway-management experience (>100 endotracheal intubations). A successfully secured airway was defined as placement of a cuffed endotracheal tube below the vocal cords.

Operators, Devices, and Airway Assessment

Airway interventions were performed by emergency physicians with substantial airway-management experience (>100 endotracheal intubations). The standardized study proforma did not capture more granular procedural variables, including the specific grade of the operator, the laryngoscopy device used at the first attempt (direct versus video laryngoscopy), the use of adjuncts such as a bougie or stylet, formal difficult-airway assessment, Cormack-Lehane glottic-view grading, or time-to-intubation metrics. These variables were therefore unavailable for analysis, as addressed in the limitations. Management after a failed first attempt followed standard institutional practice. All 244 patients were successfully intubated within two attempts, and no patient required a surgical airway. Details of the second attempt, including any change of operator, device, or adjunct, were not recorded on the study proforma.

Classification of Airway-Management Techniques

Emergency airway-management encounters were classified according to the initial technique used. RSI was defined as intubation performed after the administration of an induction agent and a neuromuscular blocking agent. DSI was defined as the use of a sedative to allow adequate preoxygenation before the subsequent stages of airway management (paralysis and intubation). Crash intubation was defined as emergency intubation performed without prior pharmacological (anesthetic) facilitation in an extremely unresponsive or unstable patient. Awake intubation was defined as tracheal intubation performed while spontaneous ventilation was maintained, without neuromuscular paralysis. For the purposes of this study, a technique referred to one airway-management strategy using a specific group of medications or airway procedures, whereas an attempt referred to a single effort to place the endotracheal tube.

The classification was mutually exclusive and is internally consistent with the medications recorded: all 38 crash intubations were performed without any pharmacological facilitation, receiving neither an induction agent nor a neuromuscular blocking agent; the 204 patients who received rocuronium comprised all RSI (n = 179) and all DSI (n = 25) cases; and the two awake intubations were performed with a sedative/induction agent but without neuromuscular blockade. The specific sedative agent, its dose, and the use of topical airway anesthesia for these two awake intubations were not recorded (see Assessment of medications used).

Study Outcomes

The primary outcomes were the first-pass success of the selected airway-management technique and the occurrence of adverse events associated with emergency airway management. First-pass success was defined as successful placement of the endotracheal tube through the vocal cords on the first intubation attempt. The secondary outcomes were the clinical indications for airway intervention and the induction and neuromuscular blocking agents used.

Assessment of adverse events

Intubation-associated adverse events occurring within 15 minutes of airway intervention were recorded. The adverse events evaluated were bradycardia, hypotension, dental trauma, airway trauma, esophageal intubation, mainstem bronchial intubation, vomiting, and cardiac arrest. Hypotension was defined as a systolic blood pressure <90 mmHg during the intubation procedure. Hypotension present before the start of airway intervention was not considered an intubation-related adverse event. Cardiac arrest was recorded as an adverse event only when it occurred during or after the initiation of airway management in a patient who was not in cardiac arrest immediately before the procedure. Adverse events in this study refer to untoward events occurring in temporal association with airway management, whereas adverse drug reactions refer specifically to noxious and unintended responses associated with the use of a medicinal product [9].

Hypoxemia was not recorded as a discrete adverse event in this study, and continuous pulse oximetry and blood pressure were not captured throughout the observation window; adverse events were ascertained by the attending airway team and entered on the study proforma immediately after the procedure. This approach is likely to under-ascertain desaturation and transient hemodynamic events relative to registries that capture them continuously, and this should be borne in mind when comparing adverse-event rates across studies. Specific physiological thresholds, such as the exact heart rate for bradycardia or a relative percentage drop in blood pressure for hypotension (beyond the absolute systolic criterion of <90 mmHg stated above), were not prospectively defined in the study protocol. Adverse events, including the severity of hypotension and the occurrence of dental trauma (e.g., visible loosening, chipping, or avulsion), were documented based on the clinical assessment of the attending emergency physician immediately following the procedure.

Clinical Indications for Airway Management

Indications for emergency airway management were classified as non-traumatic or traumatic. Non-traumatic indications evaluated were cardiac arrest, altered mental status, stroke, asthma, respiratory failure, status epilepticus, and anaphylaxis. Traumatic indications included head injury, facial trauma, and burns. Similarly, respiratory failure was classified clinically by the treating team; the data collection tool did not mandate subclassifying this into purely hypoxemic or hypercapnic failure.

Assessment of Medications Used

The induction agents recorded during airway management were ketamine, etomidate, propofol, and no induction agent. The use of neuromuscular blocking agents was also documented. Rocuronium was the only neuromuscular blocking agent recorded in this cohort, reflecting institutional availability and standard departmental protocols prioritizing its use during the study period rather than a formal study restriction. Standard institutional weight-based dosing protocols were utilized for all induction and neuromuscular blocking agents. However, the precise individual doses administered, the exact duration of preoxygenation, and the specific preoxygenation interface (e.g., non-rebreather mask versus bag-valve mask) were not granularly captured in the study dataset. Furthermore, while awake intubation was performed in two instances, the specific sedative agent, its exact dose, and the application of topical airway anesthesia were not recorded on the study proforma.

Data Collection

Data were collected prospectively using a pre-designed, pre-tested proforma that captured demographics, the indication for intubation, the airway-management technique, medications administered, the number of intubation attempts, successful intubation, and adverse events. All airway encounters were subsequently recorded on an electronic data sheet for analysis.

Statistical analysis

Categorical variables were summarized using frequencies and percentages and continuous variables using appropriate descriptive statistics (mean and standard deviation). Proportions for the primary outcomes are presented with 95% confidence intervals (CIs) calculated by the Wilson score method. Associations between categorical variables were assessed using the Fisher-Freeman-Halton exact test, which was used in preference to the chi-squared test because several expected cell counts were sparse. Airway-management techniques were compared with respect to first-pass success and adverse events. A two-sided p-value of <0.05 was considered statistically significant. Statistical analyses were performed using IBM SPSS Statistics for Windows, V. 26.0 (IBM Corp., Armonk, NY, USA).

The sample size was determined to estimate the overall first-pass success rate with a prespecified precision, not to detect differences between airway-management techniques. Because the subgroups were markedly unequal and the awake-intubation group contained only two patients, between-technique comparisons were regarded as exploratory and hypothesis-generating rather than confirmatory. Two sensitivity analyses were therefore performed for both primary outcomes: the first excluded the awake-intubation group, and the second combined the DSI and awake-intubation groups into a single alternative-technique category. No multivariable adjustment for baseline illness severity was possible because pre-intubation physiological variables were not captured in the study proforma.

Ethical Considerations

The study protocol was approved by the Academy of Medical Sciences Ethical Committee (approval number: 269/2019/ACME). Participation did not involve any additional cost to the patients. The authors declare no conflicts of interest. Given the time-critical nature of emergency airway management, where patients are typically incapacitated by critical illness (e.g., cardiac arrest, severe respiratory failure, or altered mental status), the Institutional Ethical Committee approved a deferred consent pathway. Initial life-saving interventions were performed immediately under the doctrine of presumed consent. Written informed consent for study inclusion was subsequently obtained from the patient's legally authorized representative or from the patient once clinical stability and decision-making capacity were regained. If consent was declined or could not be obtained, the patient was excluded from the final analysis; three patients were excluded on this basis. This observational study was conducted following institutional ethical approval; prospective clinical trial registration was not applicable, as no experimental interventions were allocated.

## Results

Study population and demographic characteristics

A total of 247 patients requiring airway management in the ED were assessed during the study period. Three patients could not be included because consent could not be obtained, leaving 244 patients for analysis. The mean age was 63.1 ± 9.7 years. Because the exact median and interquartile range were not extractable from the primary dataset, the age distribution is reported categorically. Most patients were aged 58-67 years (101, 41.4%), followed by 68-78 years (85, 34.8%) and 48-57 years (50, 20.5%); 236 (96.8%) patients were older than 47 years, indicating a predominantly elderly cohort. There were 136 (55.7%) male and 108 (44.3%) female participants, and males outnumbered females, as detailed in Table 1.

**Table 1:** Demographic characteristics of patients requiring emergency airway management SD: standard deviation

Characteristic	Number (n)	Percentage (%)
Age group (years)		
18-27	1	0.4
28-37	4	1.6
38-47	3	1.2
48-57	50	20.5
58-67	101	41.4
68-78	85	34.8
Total	244	100.0
Mean age ± SD	63.1 ± 9.7 years	-
Sex		
Male	136	55.7
Female	108	44.3

Distribution of Advanced Airway-Management Techniques

RSI was the most frequently used airway-management technique (179/244, 73.4%). Crash intubation was performed in 38 patients (15.6%), DSI in 25 (10.2%), and awake intubation in two (0.8%). No surgical airway procedures were required in the study population. As shown in Figure 2, the majority of airway interventions were performed using RSI, with only a small number of awake intubations.

**Figure 2:**
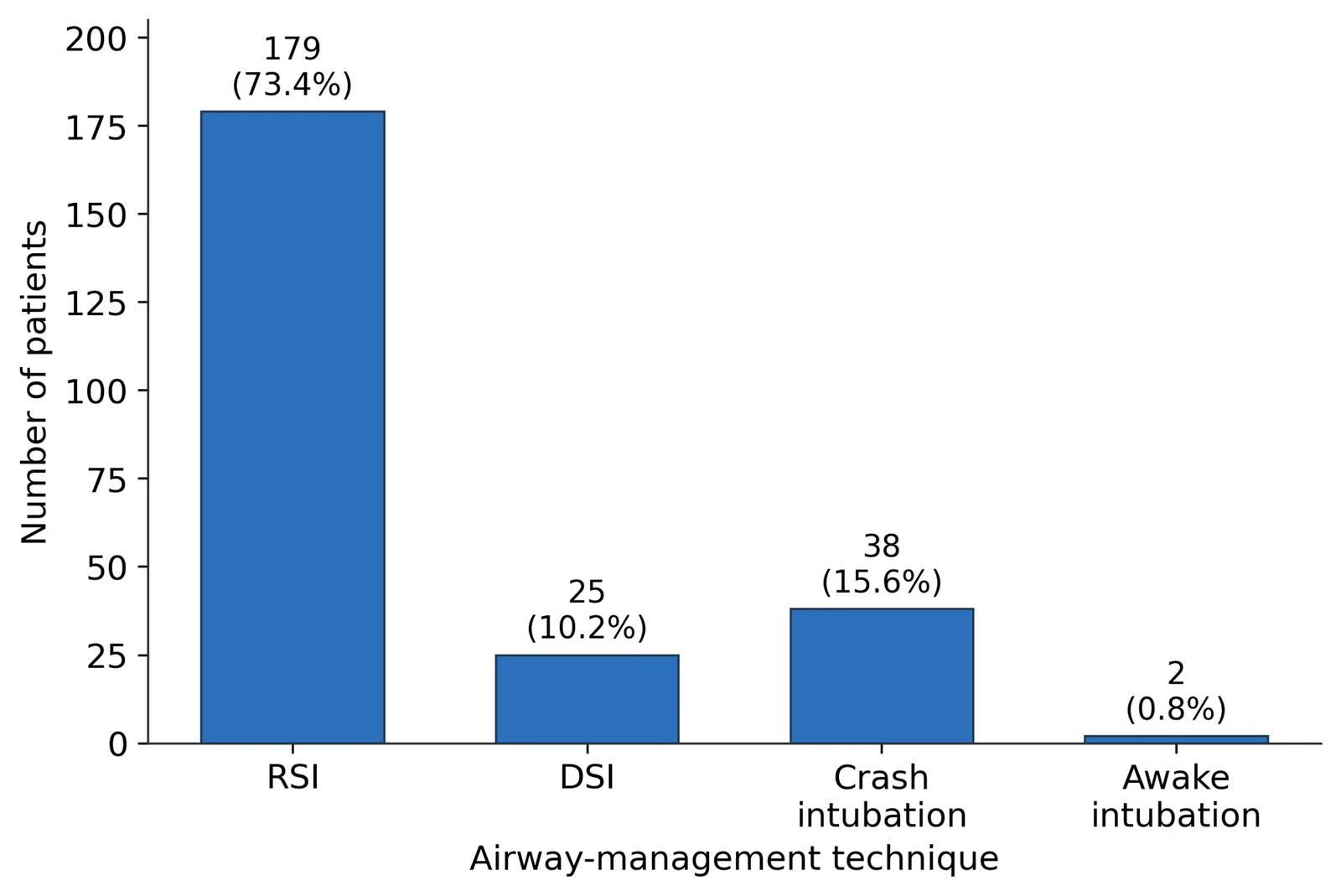
Distribution of advanced airway-management techniques RSI: rapid sequence intubation; DSI: delayed sequence intubation

First-Pass Success According to Airway Technique

Overall, 223/244 patients (91.4%; 95% CI: 87.2-94.3) had a successful first intubation attempt, and 21 patients (8.6%) required a second attempt. All 244 patients were ultimately intubated within two attempts, and no surgical airway was required. First-pass success was observed in 164 of 179 RSI cases (91.6%; 95% CI: 86.6-94.9), all 25 DSI cases (100.0%; 95% CI: 86.7-100), 32 of 38 crash intubations (84.2%; 95% CI: 69.6-92.6), and both awake-intubation cases (100.0%; 95% CI: 34.2-100). No statistically significant difference in first-pass success was observed among the four techniques (Fisher-Freeman-Halton exact test; p = 0.173). The CIs around the DSI and awake-intubation estimates are wide, and for the two awake intubations, the interval extends from 34.2% to 100.0%; these subgroup estimates are therefore highly imprecise, and the comparison is inconclusive rather than demonstrating equivalence between techniques. In sensitivity analyses, the comparison remained non-significant both when the awake-intubation group was excluded (p = 0.099) and when DSI and awake intubation were combined into a single alternative-technique category (p = 0.091). As shown in Table 2, the apparent differences in first-pass success among techniques were not statistically significant and should not be interpreted as technique effects.

**Table 2:** First-pass success according to airway-management technique P-value was calculated using the Fisher-Freeman-Halton exact test, comparing first-pass success across the four airway-management techniques (Fisher-Freeman-Halton exact test; p = 0.173). RSI: rapid sequence intubation; DSI: delayed sequence intubation

Technique	First attempt, n (%)	Second attempt, n (%)	Total	P-value
RSI	164 (91.6)	15 (8.4)	179	-
DSI	25 (100.0)	0 (0.0)	25	-
Crash intubation	32 (84.2)	6 (15.8)	38	-
Awake intubation	2 (100.0)	0 (0.0)	2	-
Overall	223 (91.4)	21 (8.6)	244	0.173

Adverse Events Associated With Emergency Airway Management

At least one airway-management-related adverse event was recorded in 30 patients (12.3%) during the observation period, while 214 patients (87.7%) had no recorded adverse events. Each affected patient experienced a single recorded adverse event; therefore, the number of adverse events (30) corresponded to the number of patients with an adverse event. Dental trauma was the most frequent event (7/30, 23.3% of all adverse events). Five patients had cardiac arrest, four had vomiting, and four had airway trauma; three patients each experienced hypotension, esophageal intubation, and mainstem intubation, and one patient had recorded bradycardia. Procedural trauma and cardiovascular events were substantial contributors to the adverse-event burden, as shown in Table 3.

**Table 3:** Distribution of adverse events associated with emergency airway management

Adverse event	Number (n)	% of all adverse events	% of total study population
Bradycardia	1	3.3	0.4
Hypotension	3	10.0	1.2
Dental trauma	7	23.3	2.9
Airway trauma	4	13.3	1.6
Esophageal intubation	3	10.0	1.2
Mainstem intubation	3	10.0	1.2
Vomiting	4	13.3	1.6
Cardiac arrest	5	16.6	2.0
Total adverse events	30	100.0	12.3

Comparison of Adverse Events Among Airway Techniques

The rate of adverse events varied among techniques. Adverse events occurred in 16 of 179 RSI cases (8.9%; 95% CI: 5.6-14.0), seven of 25 DSI cases (28.0%; 95% CI: 14.3-47.6), and seven of 38 crash intubation cases (18.4%; 95% CI: 9.2-33.4); no adverse events occurred in the two awake-intubation cases (0.0%; 95% CI: 0-65.8). There was a statistically significant association between airway-management technique and the occurrence of adverse events (Fisher-Freeman-Halton exact test; p = 0.031), which persisted in sensitivity analyses excluding the awake-intubation group (p = 0.012) and combining DSI with awake intubation (p = 0.019). As shown in Table 4, DSI had the highest proportion of adverse events (28.0%), followed by crash intubation (18.4%) and RSI (8.9%). Because the airway technique was selected on clinical grounds rather than randomly allocated, and because pre-intubation physiological severity was not recorded, this association cannot be attributed to the technique itself; it is most plausibly explained by confounding by indication, since patients managed with DSI or crash intubation were by definition more agitated, hypoxemic, or hemodynamically unstable at presentation.

**Table 4:** Adverse events according to airway-management technique P-value was calculated using the Fisher-Freeman-Halton exact test, comparing the occurrence of adverse events across the four airway-management techniques (Fisher-Freeman-Halton exact test; p = 0.031). RSI: rapid sequence intubation; DSI: delayed sequence intubation

Adverse event	RSI (n = 179)	DSI (n = 25)	Crash intubation (n = 38)	Awake intubation (n = 2)	P-value
Bradycardia	1	0	0	0	-
Hypotension	2	0	1	0	-
Dental trauma	4	2	1	0	-
Airway trauma	3	1	0	0	-
Esophageal intubation	3	0	0	0	-
Mainstem intubation	3	0	0	0	-
Vomiting	0	4	0	0	-
Cardiac arrest	0	0	5	0	-
No adverse event	163	18	31	2	-
Any adverse event	16 (8.9%)	7 (28.0%)	7 (18.4%)	0 (0.0%)	0.031

Clinical Indications for Emergency Airway Management

Of the 244 patients, 183 (75.0%) underwent airway management for non-traumatic indications and 61 (25.0%) for traumatic indications. Among non-traumatic cases, respiratory failure was the most common indication (49/183, 26.8%), followed by cardiac arrest (38/183, 20.8%), altered mental status (36/183, 19.7%), and status epilepticus (24/183, 13.1%). The most common traumatic indication was head injury (37/61, 60.7%), followed by facial trauma and burns (12 cases each, 19.7%). As shown in Table 5, three-quarters of airway interventions were for non-traumatic indications.

**Table 5:** Clinical indications for emergency airway management

Indication	Number (n)	Percentage within category (%)
Non-traumatic indications (n = 183)		
Respiratory failure	49	26.8
Cardiac arrest	38	20.8
Altered mental status	36	19.7
Status epilepticus	24	13.1
Stroke	12	6.6
Asthma	12	6.6
Anaphylaxis	12	6.6
Traumatic indications (n = 61)		
Head injury	37	60.7
Facial trauma	12	19.7
Burns	12	19.7

Induction and Neuromuscular Blocking Agents

Of the 244 patients, 206 (84.4%) received an induction agent, and 38 (15.6%) did not. The most commonly used induction agent was etomidate (84 patients, 34.4%), followed by ketamine and propofol (61 patients each, 25.0%). Neuromuscular blockade was used in 204 patients (83.6%), with rocuronium being the only agent recorded. As shown in Table 6, etomidate was the most frequently used induction agent, and rocuronium accounted for all neuromuscular blocking agent use.

**Table 6:** Pharmacological agents used during emergency airway management

Drug category	Agent	Number (n)	Percentage (%)
Induction agent	Ketamine	61	25.0
	Etomidate	84	34.4
	Propofol	61	25.0
	No induction agent	38	15.6
Neuromuscular blocking agent	Rocuronium	204	83.6
	No paralytic agent	40	16.4

## Discussion

In this prospective observational study, the use, first-pass success, and adverse events of four advanced airway-management techniques were assessed in 244 adult patients requiring emergency airway management. The overall first-pass success rate was 91.4%, and RSI was the most commonly used technique (73.4%), followed by crash intubation (15.6%), DSI (10.2%), and awake intubation (0.8%). The overall adverse-event rate was 12.3%. These findings support the central role of RSI in emergency airway management and underscore the importance of considering patient condition and clinical context when selecting a technique.

This is in keeping with international airway registries, which report RSI as the predominant initial technique for ED intubation, although its use varies with local training, protocols, and resources; RSI use is high in North America, Europe, and Australasia but remains comparatively low in some Asian settings such as Japan [1,2]. The proportion observed here (73.4%) falls within this range. The overall first-pass success rate of 91.4% is comparable to, or higher than, that of contemporary registries, in which first-pass success ranges from approximately 74.0% to 94.0% depending on setting, technique, and device [1,10]. All intubations were performed by physicians with extensive airway experience, which may have contributed to the high success rate observed.

First-pass success is clinically important because repeated attempts increase the risk of complications; an increasing number of intubation attempts have been associated with a higher incidence of peri-intubation adverse events [3]. Contemporary studies also describe strategies to maximize first-pass success, including video laryngoscopy, careful device selection, and adequate preparation [10,11]. The high first-pass success observed here is therefore a clinically meaningful strength, although it was not analyzed in detail by device in the present study.

DSI was associated with 100.0% first-pass success in this cohort, but this must be interpreted with caution. DSI is typically reserved for agitated or hypoxemic patients who cannot initially be adequately preoxygenated. Randomized data indicate that, before the onset of paralysis, oxygenation can be improved using DSI with a dissociative dose of ketamine [5]. Given the small, highly selected DSI subgroup in this study, the observed success is specific to those cases and does not indicate superiority over RSI. Only two patients underwent awake intubation; although both were intubated on the first attempt, the 95% CI around this estimate extends from 34.2% to 100.0%, so the observation carries essentially no information about the performance of the technique. Awake tracheal intubation is generally reserved for anticipated difficult airways in which spontaneous ventilation must be maintained [6]; with only two cases, no meaningful comparison with other techniques is possible, and the figure of 100.0% should not be quoted or interpreted as a success rate.

The overall adverse-event rate of 12.3% was lower than the 16.0% to 19.0% reported by contemporary emergency airway registries [12,13] and substantially lower than the 45.2% major adverse-event rate reported by the INTUBE study [3]. This difference is most likely methodological rather than a genuine indication of safer practice. First, the definitions differ: INTUBE counted major peri-intubation events including severe hypoxemia and cardiovascular instability, whereas hypoxemia was not recorded as a discrete adverse event in the present study. Second, the observation window here was limited to 15 minutes, whereas registries capturing events over longer periods will record more. Third, continuous pulse oximetry and blood-pressure data were not captured, so transient desaturation and hypotension that did not come to the attention of the airway team would not have been recorded; ascertainment by the treating team is also susceptible to under-reporting. Fourth, INTUBE enrolled critically ill patients in whom physiological derangement was near-universal. The present rate should therefore be read as a minimum estimate of peri-intubation adverse events rather than as evidence of a lower complication burden. The most frequent events recorded were dental trauma, cardiac arrest, airway trauma, and vomiting, with hypotension, esophageal intubation, and mainstem intubation less common; the predominance of dental trauma differs from registries in which hypoxemia and hypotension dominate, which is consistent with the exclusion of hypoxemia as a recorded event and with the absence of continuous physiological monitoring in this study. Adverse-event rates differed among techniques (28.0% for DSI, 18.4% for crash intubation, and 8.9% for RSI), but these differences must be interpreted cautiously given the small subgroups and the absence of randomization. The direction of the association is itself paradoxical, since DSI was associated with both the highest first-pass success and the highest adverse-event rate, which is difficult to reconcile with a causal effect of the technique and points instead to confounding by indication: patients selected for DSI or crash intubation were more physiologically unstable before the procedure. Causal comparisons among techniques are therefore not possible from these data.

Airway interventions were most commonly performed for non-traumatic indications (75.0%), consistent with registries in which medical indications predominate [1,13]. Non-traumatic indications were led by respiratory failure, followed by cardiac arrest and altered mental status, whereas head injury was the leading traumatic indication, illustrating the broad range of medical and trauma presentations requiring emergency airway management.

Etomidate was the most frequently used induction agent (34.4%), followed by ketamine and propofol (25.0% each), consistent with its reputation for hemodynamic stability and its wide use in emergency practice; etomidate is likewise the most commonly reported induction agent across international registries [1]. However, a recent large randomized trial found no mortality advantage of ketamine over etomidate, with a modest increase in peri-intubation cardiovascular collapse in the ketamine group [14]. The choice of induction agent should therefore be guided by the individual patient's physiology.

Rocuronium was the only neuromuscular blocking agent used (83.6%), reflecting institutional availability and departmental protocol during the study period rather than a comparative preference between agents. Comparable first-pass success and peri-intubation complication rates have been reported for rocuronium and succinylcholine [15], and current guidelines support the use of either agent depending on the clinical context [4]; the exclusive use of rocuronium here therefore reflects local practice and should not be read as evidence favoring either agent.

Overall, the study demonstrated high first-pass success and a low adverse-event rate with emergency airway management, with RSI remaining the most commonly used approach. The findings emphasize that careful pre-intubation assessment, appropriate technique selection, and a focus on achieving success on the first attempt are important elements of emergency airway management.

Strengths and limitations

This study has several strengths, including a prospective design that allowed the systematic documentation of airway interventions, medications, and complications as they occurred, the use of standard definitions for each airway technique, and the inclusion of both medical and trauma indications, which adds clinical relevance.

Several limitations should nevertheless be acknowledged. This was a single-center study in a predominantly elderly cohort (mean age 63.1 years, 96.8% older than 47 years), which limits generalizability to other settings and younger populations. The subgroups were markedly unequal (RSI (n = 179), crash intubation (n = 38), DSI (n = 25), awake intubation (n = 2)), making comparisons involving the awake-intubation group uninformative; the study was not powered to compare techniques. Airway techniques were selected on clinical grounds rather than randomly allocated, so confounding by indication cannot be excluded. Although operator experience was high (>100 previous intubations), the specific operator grade, direct versus video laryngoscopy, use of adjuncts such as bougies, difficult airway assessments, and time-to-intubation were not captured. Baseline pre-intubation physiological variables were also not recorded, precluding adjustment for illness severity. The study period overlapped the first year of the COVID-19 pandemic, and pandemic-related protocol deviations, including changes in airway operators, preoxygenation practices, and aerosol-containment measures, were not uniformly recorded and may have influenced procedural practice.

Adverse events were observed only for 15 minutes after intubation; hypoxemia was not recorded as a discrete event, and physiological data were not captured continuously, potentially resulting in the underestimation of adverse events. All intubations were performed by physicians with extensive airway experience, which may limit the applicability of the observed first-pass success to less experienced operators. Longer-term outcomes such as intensive care admission, duration of ventilation, and mortality were not collected, and the small number of events in several categories limits the precision of subgroup estimates. Despite these limitations, the study provides a systematically collected description of emergency airway practice and outcomes in a setting where prospective airway data are scarce.

## Conclusions

In this prospective observational study of advanced airway management in adult ED patients, first-pass success was high, and airway-related adverse events recorded within the immediate observation period were relatively infrequent. RSI was the predominant technique, and all procedures were performed by experienced operators.

The study was designed to estimate the overall first-pass success rate rather than to compare airway-management techniques, and it was not powered for such comparisons. Subgroups were markedly unequal, with only two awake intubations, and technique was selected according to clinical circumstances rather than randomly allocated. The differences observed in first-pass success and in adverse events among techniques are therefore inconclusive and are likely to reflect confounding by indication; they should not be interpreted as evidence of the superiority, equivalence, or relative safety of any technique. In particular, the 100.0% first-pass success recorded for DSI and awake intubation should not be read as a performance estimate, since the CI for the two awake intubations extends from 34.2% to 100.0%.

Non-traumatic indications predominated, with respiratory failure the most common medical indication and head injury the most common traumatic indication. Etomidate was the most frequently used induction agent, and rocuronium was the only recorded neuromuscular blocking agent. Taken together, these findings describe contemporary emergency airway practice in a single tertiary ED and support the importance of patient selection, airway assessment, preparation, and operator experience in achieving first-pass success. Adequately powered, prospective, multicenter studies that record device use and baseline physiological severity, adjust for confounding by indication, and follow patients beyond the immediate peri-intubation period are required before the relative effectiveness and safety of individual advanced airway-management techniques can be established.
